# Whole-transcriptome sequencing reveals a vernalization-related ceRNA regulatory network in chinese cabbage (*Brassica campestris* L. ssp. *pekinensis*)

**DOI:** 10.1186/s12864-021-08110-2

**Published:** 2021-11-13

**Authors:** Fengyan Shi, Hezi Xu, Chuanhong Liu, Chong Tan, Jie Ren, Xueling Ye, Hui Feng, Zhiyong Liu

**Affiliations:** Key Laboratory of Protected Horticulture, Shenyang Agricultural University, Ministry of Education, 120 Dongling Road, Shenhe District, 110866 Shenyang, China

**Keywords:** Chinese cabbage, Vernalization, Whole transcriptome, ceRNA, Non-coding RNA

## Abstract

**Background:**

The transition from vegetative growth to reproductive growth involves various pathways. Vernalization is a crucial process for floral organ formation and regulation of flowering time that is widely utilized in plant breeding. In this study, we aimed to identify the global landscape of mRNAs, microRNAs (miRNAs), long non-coding RNAs (lncRNAs), and circular RNAs (circRNAs) related to vernalization in Chinese cabbage. These data were then used to construct a competitive endogenous RNA (ceRNA) network that provides valuable information to better understand the vernalization response.

**Results:**

In this study, seeds sampled from the Chinese cabbage doubled haploid (DH) line ‘FT’ with or without vernalization treatment were used for whole-transcriptome sequencing. A total of 2702 differentially expressed (DE) mRNAs, 151 DE lncRNAs, 16 DE circRNAs, and 233 DE miRNAs were identified in the vernalization-treated seeds. Various transcription factors, such as WRKY, MYB, NAC, bHLH, MADS-box, zinc finger protein CONSTANS-like gene, and B3 domain protein, and regulatory proteins that play important roles in the vernalization pathway were identified. Additionally, we constructed a vernalization-related ceRNA–miRNA–target gene network and obtained 199 pairs of ceRNA relationships, including 108 DEmiRNA‒DEmRNA, 67 DEmiRNA‒DElncRNA, and 12 DEmiRNA‒DEcircRNA interactions, in Chinese cabbage. Furthermore, several important vernalization-related genes and their interacting lncRNAs, circRNAs, and miRNAs, which are involved in the regulation of flowering time, floral organ formation, bolting, and flowering, were identified.

**Conclusions:**

Our results reveal the potential mRNA and non-coding RNAs involved in vernalization, providing a foundation for further studies on the molecular mechanisms underlying vernalization in Chinese cabbage.

**Supplementary Information:**

The online version contains supplementary material available at 10.1186/s12864-021-08110-2.

## Background

Vegetative and reproductive growth are important developmental stages in the life cycles of terrestrial plants. Reproductive growth is regulated by various environmental factors and endogenous signals that induce flowering [1]. Flowering indicates the beginning of sexual reproduction and the formation of seeds and fruits. The flowering transition process is complex and regulated by the interaction of endogenous factors, including developmental age, and external environmental factors, such as temperature and photoperiod [2]. A variety of signals are generated to coordinate and regulate flowering time [2, 3]. In rice, wheat, and *Arabidopsis thaliana*, there are five regulatory modes affecting bolting and flowering (i.e., photoperiod, age, gibberellin, autonomous, and vernalization pathways), which function independently and complement each other [4–7].

Vernalization is crucial for the regulation of floral organ formation and flowering time [8]. Various genes are involved in the vernalization pathway; however, their effect on flowering time is mainly dependent on the flowering repressor, *FLOWERING LOCUS C* (*FLC*) [8]. *FLC* encodes a MADS-box transcription factor that inhibits the transcriptional activation causing floral organ transformation [9]. There are four *FLC* paralogs in *Brassica rapa*, three of which have inhibitory effects on flowering [10, 11]. *BrFLC5* is a weak regulatory gene that plays an important role in the regulation of flowering in Chinese cabbage [12]. *FLC* also inhibits many genes that encode flowering integration factors, including the *suppressor of CONSTANS overexpression 1* (*SOC1*) and *flowering locus T* (*FT*), and this inhibits flowering [13]. *Frigida* (*FRI*), located upstream of *FLC*, can mediate the vernalization process by positively regulating *FLC* [14]. The inhibition or mutation of either *FRI* or *FLC* leads to an early flowering phenotype [14]. The genes responsible for histone modifications of *FLC* chromatin during *Arabidopsis thaliana* bolting and flowering mainly include *vernalization insensitive 3* (*VIN3*), *vernalization 1* (*VRN1*), and *vernalization 2* (*VRN2*) [14]. The vernalization of *B. rapa* also regulates the expression of *VRN1*, *VRN2*, and *VIN3* [15]. The expression of *VIN3* is reportedly weak at room temperature, and its expression level gradually increases during vernalization and low-temperature treatments. *VIN3* can only be expressed after a long period of low-temperature induction; it inhibits the expression of *FLC* and promotes plant flowering [16]. *VRN1* encodes a DNA-binding protein that can regulate MADS-box transcription; VRN2 regulates gene expression by modifying the chromatin structure. The main function of *VRN1* and *VRN2* is to maintain the inhibitory effect of *VIN3* on *FLC* [17, 18]. Considering years of research, genes comprising the *FLC*, *VRN*, and *VIN3* families are the most thoroughly studied vernalization-related genes in Chinese cabbage [19].

When combined with transcriptomic technology, the analysis of genome-wide differential RNA expression can reveal changes in plant transcriptome levels, thus facilitating research on the molecular mechanisms underlying plant development [20, 21]. Various vernalization-related genes have been identified using transcriptome sequencing [22]. For instance, Li et al. [23] studied the whole-genome interaction dynamics and vernalization regulatory pathway of transcription factors during oriental lily development via transcriptome sequencing. In orchardgrass, the roles of the MADS-box, WRKY, bHLH, NAC, AP2/EREBP, and CCAAT families in vernalization and flower bud development were explored using RNA-Seq [24]. A series of vernalization-related genes, including two *FLC* genes, were screened during a genome-wide transcriptome analysis of radish; 775 vernalization-related candidate genes were identified according to their expression profiles [25]. Transcriptome sequencing showed that the expression patterns of *VIN1*, *trehalose-phosphate synthase* (*TPS*), *cyclic dof factor* (*CDF*), *UTP-glucose-1-phosphate uridylyltransferase* (*UGP*), and seven hormone (auxin, gibberellin, ethylene, abscisic acid, jasmonic acid, cytokinin and salicylic acid) pathway genes differed during vernalization in *B. rapa* [26]. In addition to mRNAs, cells also contain a variety of non-coding RNAs (ncRNAs), such as microRNAs (miRNAs), long non-coding RNAs (lncRNAs), and circular RNAs (circRNAs). Studies have shown that ncRNAs (COOLAIR, COLDWRAP, and COLDAIR) formed by antisense transcripts induced by low temperature play a regulatory role in the vernalization pathway mediating *Arabidopsis thaliana* bolting and flowering [27–29]. Analysis of the lncRNAs of *B. rapa* under vernalization conditions demonstrated that most of the differentially expressed lncRNAs (DElncRNAs) did not change the expression levels of the mRNAs covering or close to the lncRNAs, indicating that the transcription reactions for lncRNAs and mRNAs were independent after cold treatment. However, some differentially expressed mRNAs (DEmRNAs) have the same expression pattern as natural antisense transcripts [30]. Interactions among mRNAs, miRNAs, lncRNAs, and circRNAs can affect gene expression, and the competitive endogenous RNA (ceRNA) hypothesis has been proposed [31]. The ceRNA network has been constructed and explored in many plants, showing that it plays important roles in plant growth and development [25, 32, 33]. However, the regulatory network for ceRNAs in the vernalization pathway has not yet been established. Hence, it is important to better understand the interactions between mRNAs, miRNAs, lncRNAs, and circRNAs in the bolting and flowering of Chinese cabbage.

Chinese cabbage (*Brassica campestris* L. ssp. *pekinensis*), which originated in China, is a diploid vegetable crop of the genus *Brassica* belonging to the Cruciferae family; it is palatable, nutritious, storable, and popular among consumers [34]. Chinese cabbage is a biennial plant with seed vernalization; it is usually planted in autumn when the temperature drops from August to November in Japan and China, and it bolts and flowers in spring. One main factor affecting the yield of Chinese cabbage is temperature; low temperatures act on germinating seeds and seedlings to induce flower bud differentiation and early bolting, thus reducing their commercial value [35]. However, rapid bolting and flowering can also be beneficial when they are utilized for rapid breeding to develop improved cultivars. Hence, vernalization has positive effects on Chinese cabbage cultivation; however, further research is required to understand these effects. Moreover, it is crucial that the molecular mechanisms involved in the vernalization of Chinese cabbage are further investigated to address the current production problems.

In this study, we aimed to identify the expression patterns of mRNAs, miRNAs, lncRNAs, and circRNAs related to vernalization in Chinese cabbage to construct a ceRNA network that provides a foundation for the future exploration of the underlying regulatory mechanisms.

## Results

### Identification and functional enrichment analysis of DEmRNAs between non-vernalized (Nor) and vernalized (Ver) samples

**Fig. 1:**
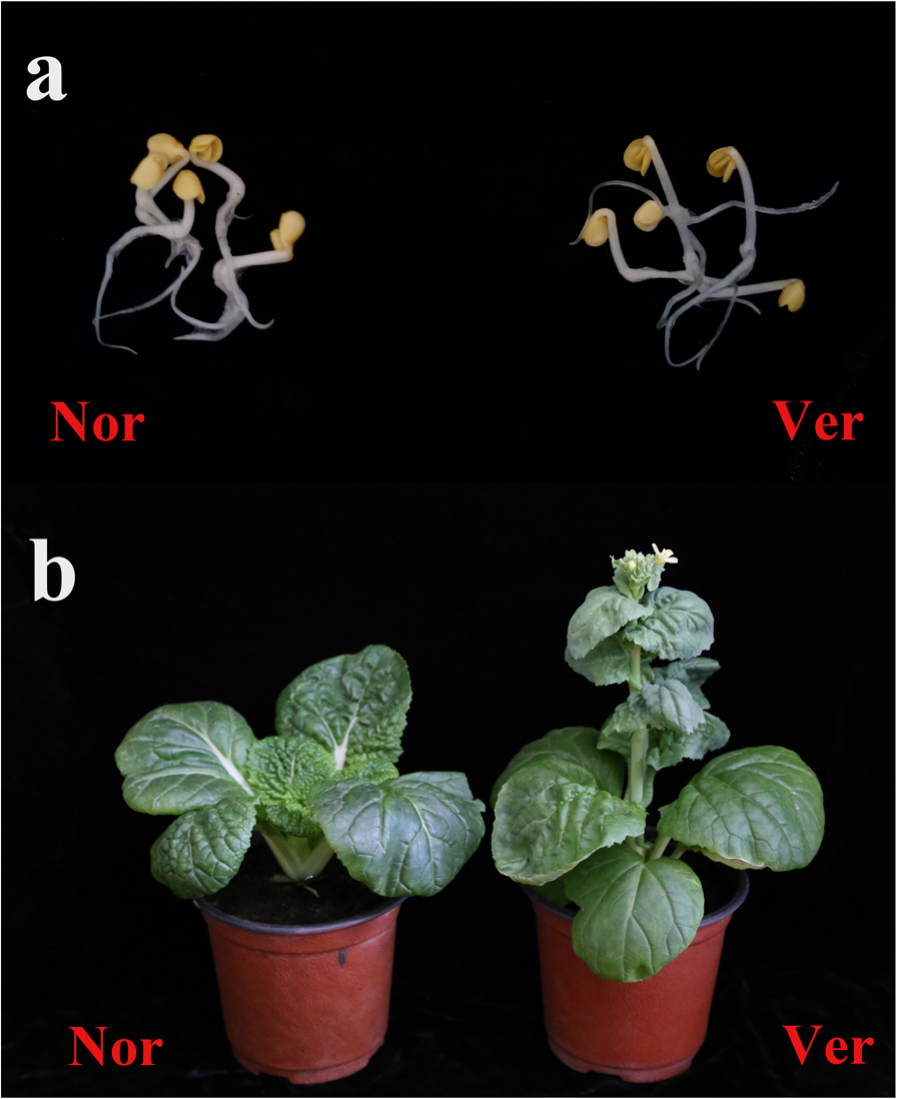
Germinated seeds (**A**) and plants (**B**) of Chinese cabbage exposed to non-vernalized (Nor) and vernalized (Ver; 4 °C for 25 d) conditions

**Fig. 2:**
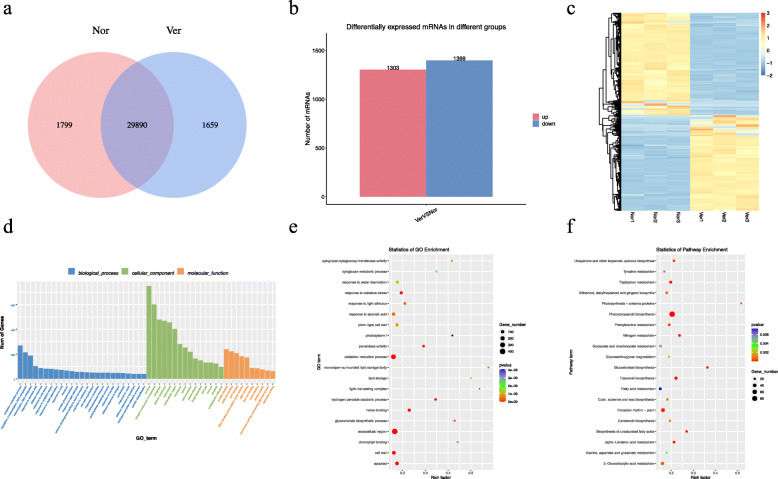
Identification and analysis of mRNAs under vernalization. **A** Venn diagram showing the number of mRNAs in non-vernalized (Nor) and vernalized (Ver) Chinese cabbage seeds; (**B**) statistical analysis of the number of up- and downregulated differentially expressed mRNAs (DEmRNAs) identified between the Nor and Ver samples; (**C**) heat map; (**D**) Gene Ontology (GO) classifications; (**E**) GO enrichment analysis; and (**F**) Kyoto Encyclopedia of Genes and Genome (KEGG) pathway assignments for all of the DEmRNAs

This study used germinated non-vernalized and vernalized (4 ℃ for 25 days) Chinese cabbage seeds (Fig. 1A). The vernalized plants were found to bolt earlier than the non-vernalized plants (Fig. 1B). Whole-transcriptome sequencing of six RNA libraries (Nor 1, Nor 2, Nor 3, Ver 1, Ver 2, and Ver 3) was performed using the Illumina Novaseq 6000 platform. A total of 318,067,362 and 308,406,152 raw reads and 260,237,854 and 259,839,298 valid reads after filtering were obtained from the Nor and Ver libraries, respectively (Table S1). After stringent filtering, the valid reads were mapped to the *B. rapa* v3.0 reference genome. A total of 33,348 mRNAs were identified; 1799 were specifically expressed in the Nor library and 1659 in the Ver library (Table S2, Fig. 2A). A | log_2_ (fold change) | > 1 and *p* ≤ 0.05 were used as the standard thresholds for screening the DEmRNAs. A total of 2702 DEmRNAs were obtained, of which 1303 were upregulated (48.22 %) and 1399 were downregulated (51.78 %) (Table S3, Fig. 2B). The expression profiles of the DEmRNAs in each treatment were visualized using a heat map; the DEmRNAs in the Nor and Ver samples were clustered separately, whereas for each of them the three replicates were clustered together (Fig. 2C).

Among the DEmRNAs, several transcription factors and regulatory proteins that play important roles in the vernalization pathway were identified, including four WRKY transcription factors, 11 MYB transcription factors, one NAC transcription factor, eight basic helix‒loop‒helix (bHLH) transcription factors, two AP2/ERFs, 11 zinc finger protein CONSTANS-like genes, and six B3 domain-containing proteins. Additionally, *BraA02g003340.3 C* and *BraA03g004170.3 C*, which were homologous to *FLC*, were downregulated after vernalization. *BraA05g005370.3 C* encoded a MADS-box protein *SOC1* that was upregulated in the Ver samples. Two upregulated *FT* genes (*BraA09g057390.3 C* and *BraA06g013820.3 C*) and three upregulated low temperature-related genes (*BraA03g020550.3 C*, *BraA03g022330.3 C*, and *BraA03g022340.3 C*) were also identified under vernalization. Furthermore, two other *FLC* genes (*BraA10g027720.3 C* and *BraA03g015950.3 C*), three *FT* genes (*BraA02g016700.3 C*, *BraA07g031650*.3 C, and *BraA06g025510*.3 C), three *SOC1* genes (*BraA04g031640.3 C*, *BraA05g005360*.3 C, and *BraA03g023780*.3 C), two *VIN3* genes (*BraA02g012310*.3 C and *BraA03g012460*.3 C), and three *VRN1* genes (*BraA01g033970*.3 C, *BraA05g028310*.3 C, and *BraA03g038610*.3 C) were identified in this study, but they were not differentially expressed between the Nor and Ver treatments.

Gene Ontology (GO) and Kyoto Encyclopedia of Genes and Genome (KEGG) enrichment analyses were performed to explore the potential functions of the DEmRNAs (Tables S4 and S5). The majority of the DEmRNAs were annotated to the GO terms “oxidation‒reduction process,” “regulation of transcription, DNA-templated,” and “transcription” under biological process (BP); to “nucleus,” “integral component of membrane,” and “cytoplasm” under cellular component (CC); and to “protein binding,” “metal ion binding,” and “ATP binding” under molecular function (MF) (Fig. 2D). Through GO enrichment analysis, 423 GO terms with significant enrichment were screened with p-values ≤ 0.05. These terms included the oxidation‒reduction process, heme binding, extracellular region, apoplast, peroxidase activity, cell wall, response to oxidative stress, glucosinolate biosynthetic process, monolayer-surrounded lipid storage body, and response to light stimulus (Fig. 2E), which may be involved in the regulation of vernalization in Chinese cabbage. According to the KEGG enrichment analysis, these DEmRNAs were enriched in 132 pathways, including 36 significantly enriched KEGG metabolic pathways (*p* ≤ 0.05) (Fig. 2F). Among them, the pathways “photosynthesis‒antenna proteins” and “circadian rhythm‒plant” were significantly enriched in the Nor and Ver samples and played an important role in flowering regulation.

### Identification and functional enrichment analysis of DElncRNAs between the Nor and Ver samples

**Fig. 3:**
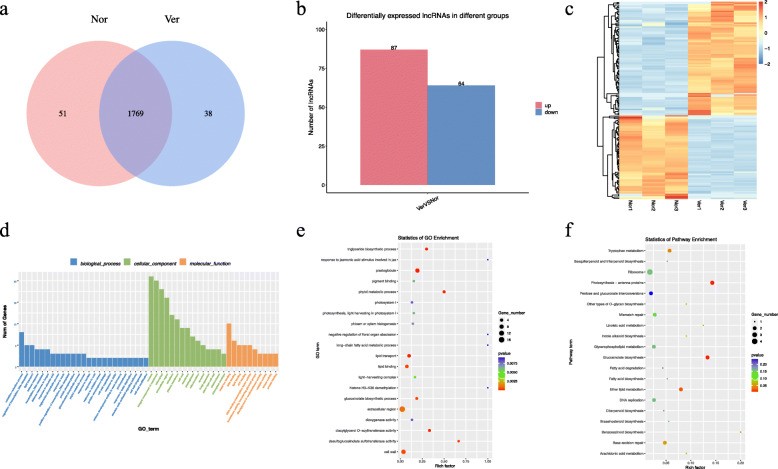
Identification and analysis of long-coding RNAs (lncRNAs) under vernalization. **A** Venn diagram showing the number of lncRNAs in non-vernalized (Nor) and vernalized (Ver) Chinese cabbage seeds; (**B**) statistics for the number of up- and downregulated differentially expressed lncRNAs (DElncRNAs) identified between the Nor and Ver samples; (**C**) heat map; (**D**) Gene Ontology (GO) classifications; (**E**) GO enrichment; and (**F**) Kyoto Encyclopedia of Genes and Genome (KEGG) pathway assignments for all DElncRNAs

In addition to the mRNAs, a total of 1858 lncRNAs were identified, of which 51 were specific to the Nor samples and 38 were specific to the Ver samples (Table S2, Fig. 3A). A total of 151 DElncRNAs were screened between Nor and Ver (| log_2_ (fold change) | > 1 and *p* ≤ 0.05). Among them, 87 DElncRNAs were upregulated (57.62 %) and 64 were downregulated (42.38 %) (Fig. 3B). The general expression profiles of these DElncRNAs are shown in Fig. 3C.

**Fig. 4:**
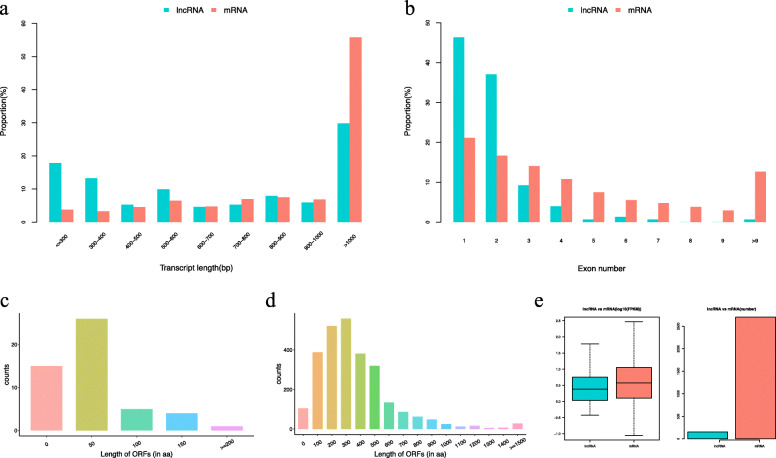
Comparison of the DElncRNA and DEmRNA structural characteristics and expression levels. **A** Transcript length distribution of DElncRNAs and DEmRNAs; (**B**) exon number of DElncRNAs and DEmRNAs; (**C** and **D**) ORF length distribution of DElncRNAs and DEmRNAs; and (**E**) DElncRNA and DEmRNA expression levels. DElncRNAs: differentially expressed long-coding RNAs; DEmRNAs: differentially expressed mRNAs

Genomic comparisons between DElncRNAs and DEmRNAs revealed that long (> 1000 bp) DEmRNAs were more abundant than long DElncRNAs whereas short (< 400 bp) DElncRNAs were more abundant than short DEmRNAs. The average open reading frame (ORF) was longer for the DEmRNAs than for the DElncRNAs. The ORFs of the DElncRNAs were predicted to be 0‒50 aa long, whereas the ORFs of the majority of the DEmRNAs were 100‒500 aa long. Furthermore, the DElncRNAs had fewer exons (1–2) than the DEmRNAs on average, and the expression levels of the DEmRNAs were higher than those of the DElncRNAs (Fig. 4).

**Fig. 5:**
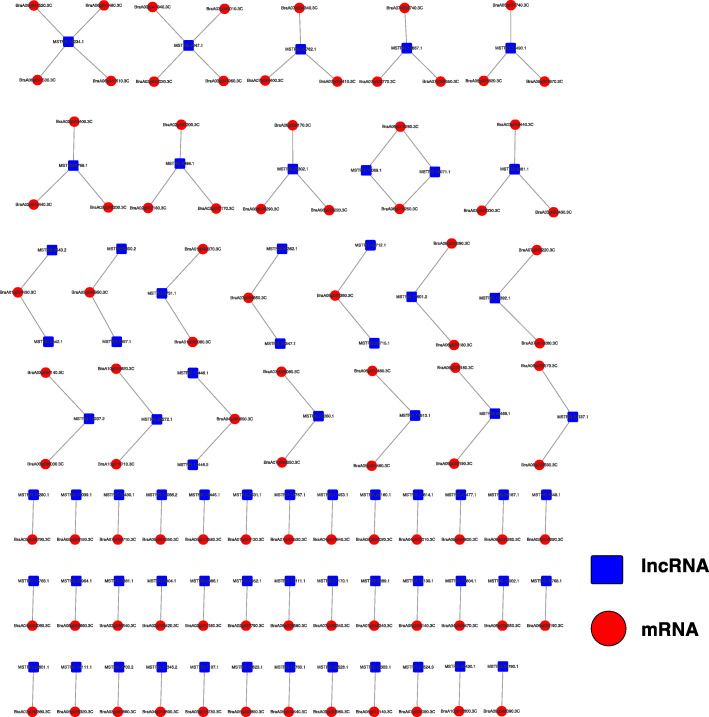
Co-expression network of differentially expressed long-coding RNAs (DElncRNAs) and differentially expressed mRNAs (DEmRNAs)

lncRNAs can cis-regulate adjacent genes, thereby regulating transcriptional or post-transcriptional gene expression [36]. The co-expression network of DElncRNAs and DEmRNAs was constructed by analyzing the cis-regulatory functions of the DElncRNAs (Fig. 5). Most of the interactions between DEmRNAs and DElncRNAs were one-to-one interactions; however, there were also one-to-many interactions.

To explore the functions of the DElncRNAs, we conducted GO and KEGG enrichment analyses of the differentially expressed genes targeted by DElncRNAs (Tables S4 and S5). The targets of the DElncRNAs were annotated in 289 GO terms, the majority of which were in “oxidation‒reduction process,” “lipid transport,” and “regulation of transcription, DNA-templated” under BP; in “nucleus,” “integral component of membrane,” and “chloroplast” under CC; and in “metal ion binding,” “DNA binding,” and “lipid binding” under MF (Fig. 3D). GO enrichment analysis of these targets showed that the plastoglobule, phytol metabolic process, diacylglycerol O-acyltransferase activity, triglyceride biosynthetic process, desulfoglucosinolate sulfotransferase activity, and glucosinolate biosynthetic process were significantly enriched terms and may be involved in vernalization regulation (Fig. 3E). For KEGG pathways, we analyzed the enrichment of the target genes of DElncRNAs and identified 46 pathways, of which five were significantly (*p* ≤ 0.05) enriched metabolic pathways (namely, photosynthesis–antenna proteins, glucosinolate biosynthesis, lipid metabolism, tryptophan metabolism, and benzoxazinoid biosynthesis) (Fig. 3F).

### Identification and functional enrichment analysis of differentially expressed circRNAs (DEcircRNAs) between Nor and Ver samples

**Fig. 6:**
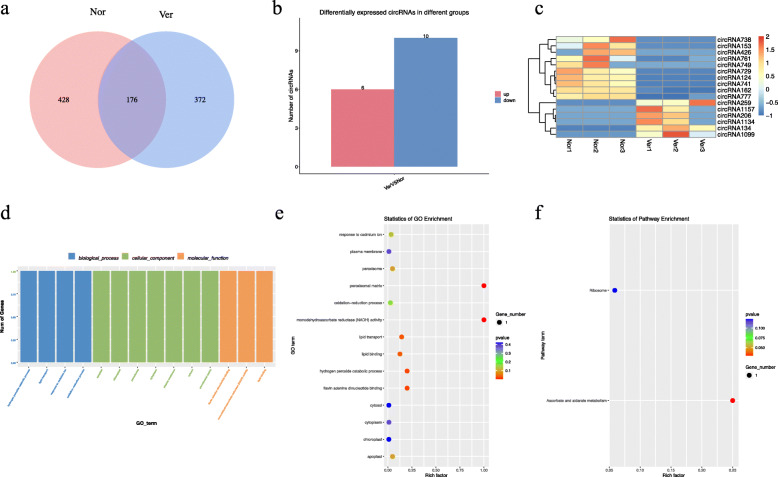
Identification and analysis of circular RNAs (circRNAs) under vernalization. **A** Venn diagram showing the number of circRNAs in non-vernalized (Nor) and vernalized (Ver) Chinese cabbage seeds; (**B**) statistics for the number of up- and downregulated differentially expressed circRNAs (DEcircRNAs) identified between the Nor and Ver samples; (**C**) heat map; (**D**) Gene Ontology (GO) classifications; (**E**) GO enrichment; and (**F**) Kyoto Encyclopedia of Genes and Genome (KEGG) pathway assignments for all DEcircRNAs

A total of 976 circRNAs were identified in the Nor and Ver samples, of which 428 were unique to Nor and 372 were unique to Ver (Table S2, Fig. 6A). After screening, a total of 16 DEcircRNAs were obtained (| log_2_ (fold change) | > 1 and *p* ≤ 0.05), including six upregulated and ten downregulated DEcircRNAs (Table S3, Fig. 6B). A heat map of DEcircRNAs illustrated the general expression profiles of DEcircRNAs in each treatment sample (Fig. 6C). To further understand the functions of DEcircRNAs, we conducted GO and KEGG enrichment analyses of the DEcircRNA-hosting genes (Tables S4 and S5). The DEcircRNA-hosting genes were annotated to 14 GO terms, and six GO terms were identified as significantly enriched (*p* ≤ 0.05). Among them, two GO terms were enriched in BP (hydrogen peroxide catabolic process and lipid transport), one in CC (peroxisomal matrix), and three in MF (monodehydroascorbate reductase [NADH] activity, flavin adenine dinucleotide binding, and lipid binding) (Fig. 6D and E). Two pathways, “ascorbate and aldarate metabolism” and “ribosomes,” were identified through KEGG enrichment analysis (Fig. 6F).

### Identification and functional enrichment analysis of differentially expressed miRNAs (DEmiRNAs) between Nor and Ver samples

**Fig. 7:**
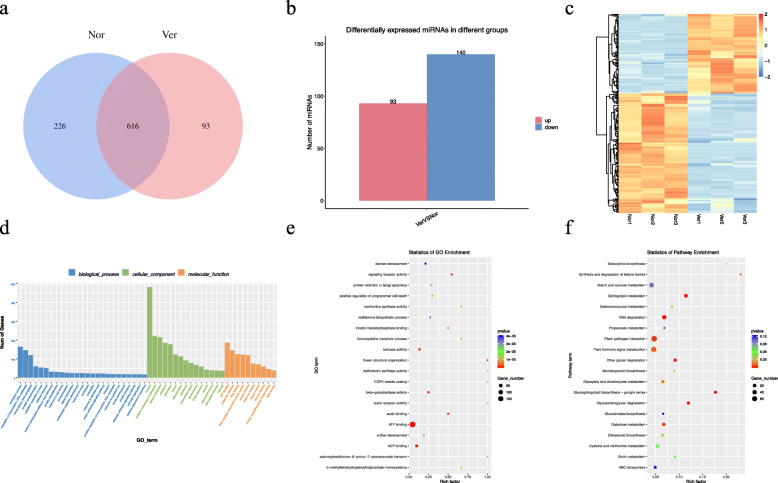
Identification and analysis of microRNAs (miRNAs) under vernalization. **A** Venn diagram showing the number of miRNAs in non-vernalized (Nor) and vernalized (Ver) Chinese cabbage seeds; (**B**) statistics for the number of up- and downregulated differentially expressed miRNAs (DEmiRNAs) identified between the Nor and Ver samples; (**C**) heat map; (**D**) Gene Ontology (GO) classifications; (**E**) GO enrichment; and (**F**), Kyoto Encyclopedia of Genes and Genome (KEGG) pathway assignments for all DEmiRNAs

To comprehensively understand the miRNA repertoire related to the vernalization of Chinese cabbage, Nor and Ver sRNA libraries were constructed and sequenced. After filtering, a total of 52,789,614 and 52,288,460 raw reads and 16,281,349 and 20,352,268 valid reads were obtained from the Nor and Ver libraries, respectively (Table S6). A total of 935 miRNAs were identified, of which 226 were specific to the Nor sample and 93 were specific to the Ver samples (Table S2, Fig. 7A). A total of 233 DEmiRNAs with (| log_2_ (fold change) | > 1 and *p* ≤ 0.05) were identified, of which 93 were upregulated (39.91 %) and 140 were downregulated (60.09 %) (Table S3, Fig. 7B). Similar to the DEmRNAs and DElncRNAs, the DEmiRNAs in the Nor and Ver samples were clustered separately, whereas in each case the three repetitions were clustered together (Fig. 7C). Additionally, the target mRNAs of these DEmiRNAs were predicted.

To further understand the potential functions of DEmiRNAs, target genes of the DEmiRNAs were investigated using GO and KEGG analyses (Tables S4 and S5, Fig. 7D‒F). The target genes of the DEmiRNAs were enriched for 1616 GO terms, 277 of which were identified as significantly enriched GO terms (*p* ≤ 0.05). Among these significantly enriched terms, 146 were enriched in BP (mainly in “regulation of transcription, DNA-templated,” “transcription,” and “protein phosphorylation”), 33 in CC (mainly in “nucleus,” “plasma membrane,” and “chloroplast envelope”), and 98 in MF (mainly in “ATP binding,” “DNA binding,” and “DNA-binding transcription factor activity”). Among them, we found that some GO terms were related to vernalization and floral organ development (such as flower structural organization, floral organ abscission, floral organ senescence, and flower development). Further, we performed KEGG enrichment analysis of the target genes of DEmiRNAs and identified 115 pathways. Several pathways involved in the vernalization and flowering regulation process, including the “starch and sucrose metabolism,” “circadian rhythm‒plant,” and “carbon fixation in photosynthetic organisms” pathways, were also identified. Twelve KEGG metabolic pathways were found to be significantly enriched (*p* ≤ 0.05).

### ceRNA network construction

**Fig. 8:**
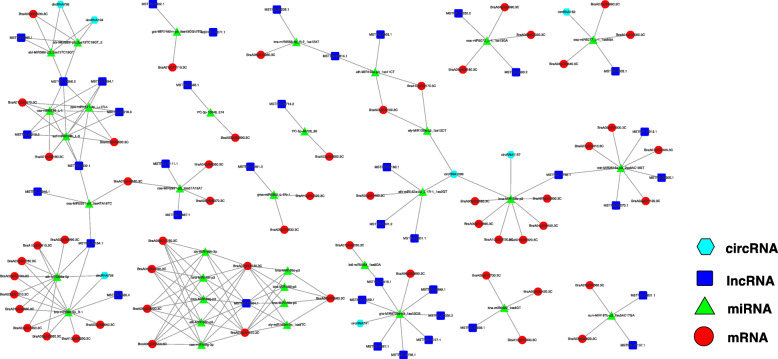
Competitive endogenous RNA (ceRNA) network constructed with all differentially expressed mRNAs (DEmRNAs), differentially expressed long-coding RNAs (DElncRNAs), differentially expressed circular RNAs (DEcircRNAs), and differentially expressed microRNAs (DEmiRNAs)

lncRNAs and circRNAs can interact with miRNAs through miRNA response elements in the ceRNA network [37]. To reveal the global regulatory network of protein-coding RNAs and ncRNAs that are related to vernalization, ceRNA networks were constructed based on the ceRNA theory using DEmRNAs, DEmiRNAs, DElncRNAs, and DEcircRNAs. We established candidate ceRNA relationships using miRNA–target relationships and obtained 199 pairs of ceRNA relationships, including 108 DEmiRNA‒DEmRNA, 67 DEmiRNA‒DElncRNA, and 12 DEmiRNA‒DEcircRNA interactions (Table S7). A Perl script was used to integrate lncRNA–miRNA–mRNA and circRNA–miRNA–mRNA networks based on the relationships among the miRNA–mRNA, lncRNA–miRNA, and circRNA–miRNA interactions. Cytoscape software (https://cytoscape.org) was used to visualize the regulatory relationships (Fig. 8). According to the functional descriptions of the genes, we identified various known DEmRNAs that were directly or indirectly related to vernalization from the ceRNA networks; these included 2-oxoglutarate-dependent dioxygenase (*BraA06g010260.3 C* regulated by bra-miR9556-5p, *MSTRG.22206.1*, and *MSTRG.28814.1*), E3 ubiquitin-protein ligase (*BraA04g006360.3 C* regulated by osa-miR2931-p3, *MSTRG.17111.1*, and *MSTRG.19887.1*), F-box protein (*BraA03g003100.3 C* regulated by ath-MIR159a-p3, *MSTRG.5303.1*, *MSTRG.28814.1*, and *circRNA1099* and *BraA01g026330.3 C* regulated by bdi-miR5054 and *MSTRG.15419.1*), growth-regulating factor (*BraA03g025790.3 C*, *BraA03g061010.3 C*, *BraA09g043950.3 C*, and *BraA02g014450.3 C* regulated by miR396a-5p, *MSTRG.25184.1*, *MSTRG.7620.4*, and *circRNA729*), low temperature-induced protein (*BraA03g020550.3 C* regulated by PC-5p-49728_80 and *MSTRG.26714.2*), methyltransferase (*BraA07g034410.3 C* and *BraA07g034400.3 C* regulated by mtr-MIR2615a-p3, *MSTRG.11766.1*, *MSTRG.14913.1*, *MSTRG.25305.1*, and *MSTRG.25070.1*), serine/threonine-protein kinase (*BraA01g033070.3 C* regulated by miR5139, *MSTRG.5632.1*, *MSTRG.10356.2*, *MSTRG.29218.2*, *MSTRG.29218.3*, and *MSTRG.5494.1*), protein phosphatase 2 C (*BraA03g001030.3 C* regulated by miR169 and *MSTRG.22964.1*), WD repeat-containing protein (*BraA04g026690.3 C* regulated by osa-miR5072, *MSTRG.9400.2*, and *MSTRG.19252.2*), and zinc finger protein (*BraA10g023720.3 C* regulated by bna-MIR156b-p3, *MSTRG.11766.1*, *circRNA1157*, and *circRNA1099*).

### Verification of RNA‒Seq results by qRT‒PCR

**Fig. 9:**
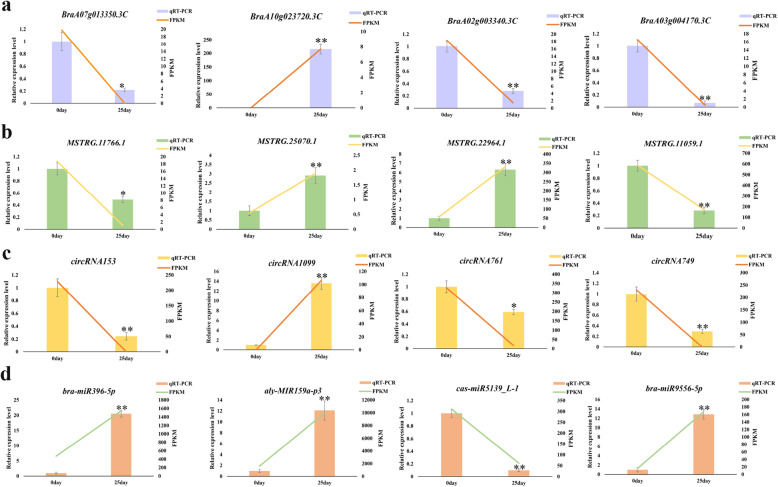
Quantitative real-time polymerase chain reaction (qRT‒PCR) analysis. qRT-PCR results for (**A**) differentially expressed mRNAs (DEmRNAs); (**B**) differentially expressed long-coding RNAs (DElncRNAs); (**C**) differentially expressed circular RNAs (DEcircRNAs); and (**D**) differentially expressed microRNAs (DEmiRNAs) in non-vernalized (Nor) and vernalized (Ver) Chinese cabbage seeds. **P < 0.01 and *P < 0.05, based on Duncan’s test

**Fig. 10:**
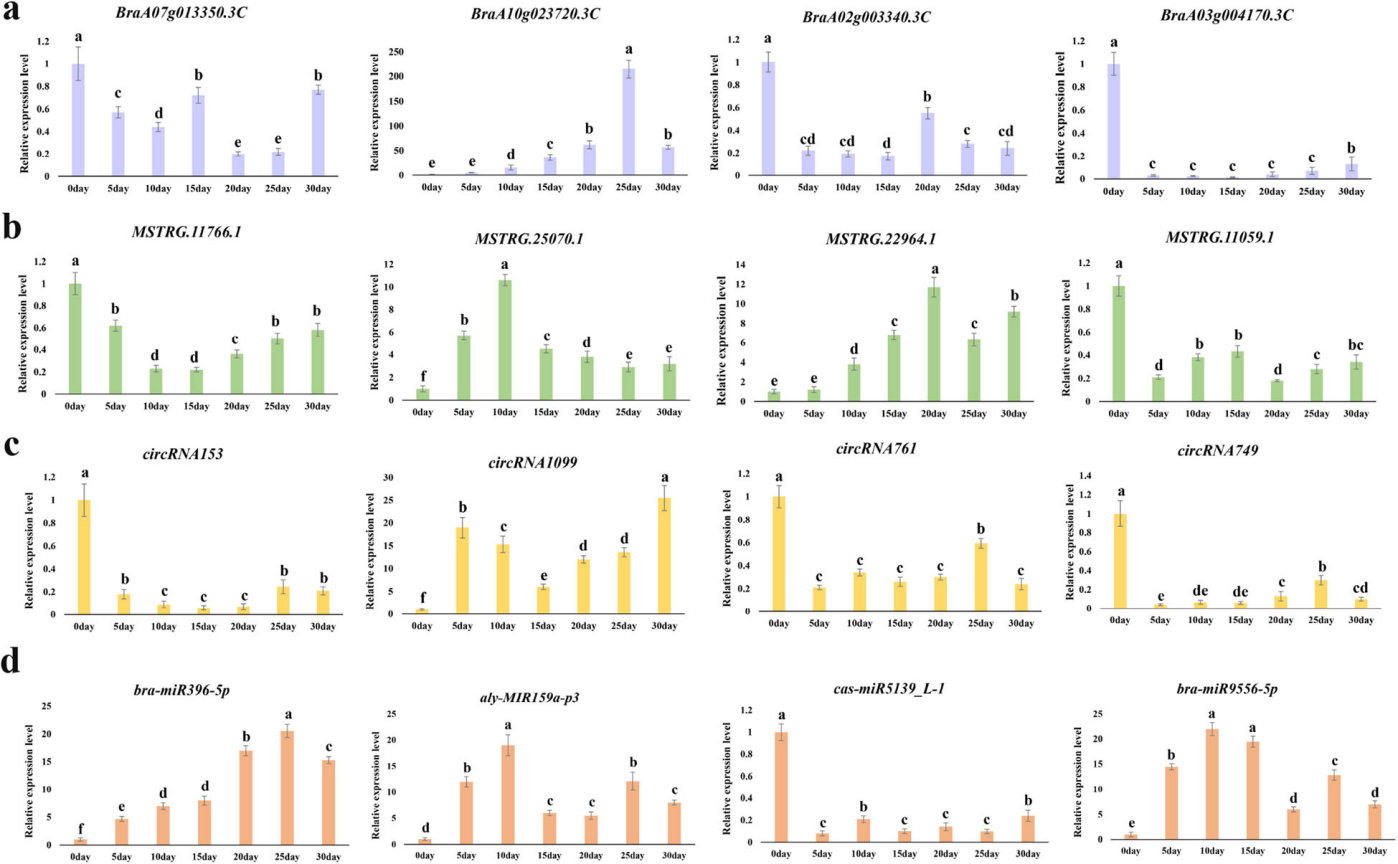
Quantitative reverse transcriptase-polymerase chain reaction (qRT‒PCR) analysis. qRT-PCR results for the (**A**) differentially expressed mRNAs (DEmRNAs); (**B**) differentially expressed long-coding RNAs (DElncRNAs); (**C**), differentially expressed circular RNAs (DEcircRNAs); and (**D**) differentially expressed microRNAs (DEmiRNAs) in Chinese cabbage seeds treated with different vernalization times. Different letters indicate significant differences among the different treatments according to the least significant difference (LSD) test at *P* < 0.05

To verify the reliability of the RNA‒Seq results, we randomly selected four DEmRNAs, four DEmiRNAs, four DElncRNAs, and four DEcircRNAs for qRT‒PCR. The expression patterns of these RNAs were concordant with the RNA‒Seq data, suggesting that the RNA‒Seq results were reliable (Fig. 9). Among them, *BraA02g003340.3 C* and *BraA03g004170.3 C* are homologous to *FLC* in Chinese cabbage. The experimental results showed that the expression of these two genes was downregulated after vernalization, which was consistent with previously reported results [38]; this further proves the accuracy of the experimental and sequencing results. We then analyzed the expression patterns of the 16 differentially expressed RNAs under different vernalization treatments (i.e., vernalization at 0, 5, 10, 15, 20, 25, and 30 days). Each RNA had its own different expression pattern, indicating that the degree of vernalization differed, as did the expression levels of the four types of RNA (Fig. 10).

## Discussion

Vernalization is a key process in premature bolting and flowering, and the transition from vegetative to reproductive growth involves multiple pathways that are critical to floral organ formation and regulation of flowering time [39]. Although the regulatory mechanism of vernalization has been gradually revealed in *Arabidopsis thaliana* [39], the molecular mechanism in Chinese cabbage vernalization remains unclear. Additionally, the comprehensive identification and analysis of ceRNA networks related to Chinese cabbage vernalization are not yet complete. It has been previously reported that lncRNAs and circRNAs (as ceRNAs) regulate each other by competitively binding to common miRNA response elements [40]. Thus, the regulatory network of ceRNAs, formed by lncRNAs, circRNAs, miRNAs, and mRNAs through miRNA response elements, is of great importance to the post-transcriptional regulation of genes in various physiological and pathophysiological processes [40]. Here, we obtained 2702 DEmRNAs, 151 DElncRNAs, 16 DEcircRNAs, and 233 DEmiRNAs by whole-transcriptome analysis between Nor and Ver samples of Chinese cabbage. We constructed the first vernalization-related ceRNA‒miRNA–target gene regulatory network to provide a basis for further study of the molecular mechanism of vernalization of Chinese cabbage.

GO enrichment and KEGG analyses were performed to explore the potential functions of DEmRNAs and the targets of DElncRNAs, DEcircRNAs, and DEmiRNAs. After low-temperature treatment, the expression patterns of many genes related to vernalization, bolting, flowering, and floral organ development change [41]. This study identified some significantly enriched GO terms associated with vernalization and floral organ development, including flower structural organization, floral organ abscission and senescence, flower development, oxidation‒reduction processes, the cell wall, responses to oxidative stress, and the glucosinolate biosynthetic process, indicating that these clusters may participate in responses to stimuli and guide vernalization. Plant hormones play an important role in the regulation of flowering time in *Arabidopsis thaliana* [42]. During the vernalization of *B. rapa*, differentially expressed genes are enriched in various hormone biosynthesis and signaling pathways [26]. In this study, 105 DEmRNAs, 5 targets of DElncRNAs, and 69 targets of DEmiRNAs were enriched in plant hormone signal transduction pathways. Sucrose and starch are the main products of photosynthetic carbon assimilation. Previous studies have shown that sucrose treatment could accelerate lily flowering and that maltose could provide *B. rapa* with enough energy for normal growth during vernalization [24, 43]. This study identified some differentially expressed RNAs and their target genes (94 DEmRNAs, 3 DElncRNAs, and 53 DEmiRNAs) that were significantly enriched in the starch and sucrose metabolism pathway, which may play a role in energy supply during the vernalization of Chinese cabbage. In addition, circadian rhythm and carbon fixation pathways were also identified, which were shown to be involved in vernalization and flowering regulation in orchardgrass and sugar beets [24, 44].

Vernalization is one of the main flowering regulation mechanisms in plants. A period of low temperature prior to flowering is required to complete vernalization, which is regulated by a series of related genes [24]. To date, *Arabidopsis thaliana* has been used as a model plant species to study the vernalization mechanism [13, 45]. Previous studies have shown that the upstream gene *FLC* has a negative regulatory effect on *FT*. The *FLC* expression level is reportedly low at the vernalization stage and then increases gradually until the bolting stage. In contrast, the expression level of *FT* is high during vernalization but low at other stages [24, 46]. The overexpression of *BrFLC3* in transgenic *B. rapa* and *BrFLC1*, *BrFLC2*, and *BrFLC3* in *Arabidopsis thaliana* delays the flowering time, indicating that these three genes are functional [47]. In the present study, *BraA02g003340.3 C* and *BraA03g004170.3 C* were found to be homologous to *FLC* in Chinese cabbage; the results of RNA‒Seq and qRT‒PCR showed that their expression was downregulated after vernalization, whereas that of the *FT* genes *BraA09g057390.3 C* and *BraA06g013820.3 C* was upregulated. These results were consistent with the previous findings that a low temperature inhibits the expression of *FLC* and reverses the inhibition of *FT*, leading to flowering transformation [38]. Additionally, three low temperature-related genes—*BraA03g020550.3 C* (low temperature-induced protein), *BraA03g022330.3 C*, and *BraA03g022340.3 C* (cold-regulated protein)—are reported to be upregulated with vernalization.

Analysis of transcription-related genes showed that transcription factors play an important role in vernalization and flower bud formation. WRKY transcription factors are involved in cell wall formation and moderate flowering [48]. The majority of MYB proteins play roles in various plant-specific developmental processes, especially in regulating flower development and stress responses [49]. Our results showed that four WRKY and 11 MYB transcription factors were differentially expressed between the Nor and Ver samples. The MADS-box family plays an important role in the evolution of flower development [50], and MADS-box transcription factors could inhibit the overexpression of *SOC1* signaling, thereby affecting short-day flowering time [51]. NAC transcription factors have been described in recent years [52] and are closely related to embryo, flower organ, and vegetative development [53]. In this study, we identified the *BraA05g005370.3 C*-encoded MADS-box protein *SOC1* and *BraA05g031600.3 C-*encoded NAC transcription factor, both of which were upregulated in the Ver samples. Previous research has demonstrated that the B3 domain-containing protein and zinc finger protein CONSTANS-like gene family are involved in early flower development and flowering regulation [54, 55]. Here, six B3 domain-containing protein genes (five upregulated and one downregulated) and 11 zinc finger protein CONSTANS-like genes (ten upregulated and one downregulated) were identified, indicating that they may play an important role in the vernalization of Chinese cabbage. The basic helix‒loop‒helix (bHLH) transcription factor has been reported to participate in the control of cell proliferation and the formation of specific cell lines [56]. Ethylene-responsive transcription factor (ERF) encodes a protein containing AP2, and various AP2/ERFs are involved in the stress response and regulation of flower development [57]. In the present study, two AP2/ERFs and eight bHLH transcription factors were differentially expressed between the Nor and Ver samples.

We initially constructed a vernalization-related ceRNA network using DEmRNAs, DEmiRNAs, DElncRNAs, and DEcircRNAs based on the ceRNA theory and obtained 199 pairs of ceRNA relationships (Fig. 8). It is well known that *FLC* is a key gene in the vernalization pathway and that it is regulated by two lncRNAs (*COLDAIR* and *COOLAIR*) [58, 59]. In this work, we did not find any vernalization-related miRNAs targeting the *FLC* genes *BraA02g003340*.3 C and *BraA03g004170*.3 C, suggesting that vernalization regulates flowering by regulating other metabolic pathways and *FLC* at the epigenetic level through miRNAs, which was in accordance with the finding of Huang et al. [60]. In the ceRNA network, we identified known DEmRNAs that play different roles in the vernalization pathway and their corresponding DEmiRNAs, DElncRNAs, and DEcircRNAs. 2-oxoglutarate-dependent dioxygenase has histone demethylation activity and can regulate *FLC* expression [61]. Protein phosphatase 2 C is involved in the regulation of flowering time [62]. In the present study, *BraA06g010260.3 C* (2-oxoglutarate-dependent dioxygenase) was found to be a target gene of bra-miR9556-5p and interacted with *MSTRG.22206.1* and *MSTRG.28814.1*, whereas *BraA03g001030.3 C* (protein phosphatase 2 C) was a target gene of miR169 and interacted with *MSTRG.22964.1.* F-box proteins regulate flower bud differentiation by promoting *FLC* expression in *Arabidopsis thaliana* [63]. The CONSTANS protein could be reversed by E3 ubiquitin-protein ligase at low temperatures to prevent early flowering [64]. Thus, *BraA04g006360.3 C* (E3 ubiquitin‒protein ligase), *BraA03g003100.3 C*, and *BraA01g026330.3 C* (F-box protein) and their related ceRNAs may be involved in the bolting and flowering of Chinese cabbage. MicroRNA396 (miR396) regulates flower organ development by targeting growth-regulating factors and participates in the vernalization and flower development of *Agrostis stolonifera* [65]. Four growth-regulating factors (*BraA03g025790.3 C*, *BraA03g061010.3 C*, *BraA09g043950.3 C*, and *BraA02g014450.3 C*) were regulated by miR396a-5p and interacted with *MSTRG.25184.1*, *MSTRG.7620.4*, and *circRNA729.* In addition, low temperature-induced proteins (*BraA03g020550.3 C)* and zinc finger protein (*BraA10g023720.3 C)*, and the corresponding miRNAs, lncRNAs, and circRNAs with which they interacted, were also identified. Further studies on the functions of these vernalization-related ceRNAs, miRNAs, and target genes will advance current understanding of the molecular mechanisms of plant bolting and flowering.

## Conclusions

In this study, whole-transcriptome sequencing was performed on Nor and Ver samples of Chinese cabbage. A total of 2702 mRNAs, 151 lncRNAs, 16 circRNAs, and 233 miRNAs were identified as being differentially expressed between the two samples. GO annotation and KEGG pathway enrichment analyses were performed on the differentially expressed RNAs, revealing their important roles in the regulation of vernalization. Several transcription factors and regulatory proteins that play important roles in the vernalization pathway were identified, such as WRKY transcription factors, MYB transcription factors, NAC transcription factors, bHLH transcription factors, MADS-box, zinc finger protein CONSTANS-like genes, and B3 domain-containing protein. Moreover, we constructed the first vernalization-related ceRNA‒miRNA–target gene regulatory network in Chinese cabbage, comprising 56 DEmRNAs, 34 DEmiRNAs, 40 DElncRNAs, and 7 DEcircRNAs, and identified several genes involved in the regulation of flowering time, floral organ formation, bolting, and flowering. Our findings provide a systematic recognition of mRNAs and ncRNAs and lay a foundation for the further exploration of the molecular regulation mechanism of vernalization in Chinese cabbage.

## Methods

### Plant materials

The double haploid line ‘FT,’ derived from Chinese cabbage variety ‘Fukuda 50,’ was obtained from a microspore culture at the Liaoning Key Laboratory of Genetics and Breeding of Cruciferous Vegetable Crops at Shenyang Agricultural University (Shenyang, China) [66]. The seeds of the control group and the treatment group were germinated and grown in wet filter paper in three culture dish at room temperature (25 ℃). Put 20 seeds in each culture dish, which is a biological repetition. After germination, the seeds were divided into two groups, one of which was vernalized. For the vernalization treatment (Ver), the germinated seeds were placed in a refrigerator at 4 ℃ for 25 d under dark conditions. The second group of seeds (Nor; without low-temperature vernalization) was stored at 25 ℃ for approximately 48 h under dark conditions. When the two groups attained the same length, the seeds were sampled and stored at − 80 °C until subsequent RNA extraction.

### RNA extraction, library construction, and RNA sequencing

Total RNA was extracted from the non-vernalized (Nor 1, Nor 2, and Nor 3) and vernalized (Ver 1, Ver 2, Ver 3) germinated seeds and treated with TRIzol reagent (Invitrogen, Carlsbad, CA, USA). RNA integrity and quality were analyzed using a NanoDrop 2000 (Thermo, DE, USA) and an Agilent 2100 Bioanalyzer (Agilent, CA, USA).

For mRNAs, lncRNAs, and circRNAs, a strand-specific library was constructed by removing ribosomal RNA using an Epicenter Ribosomal RNA Zero Gold kit (Illumina, San Diego, CA, USA). Each treatment had three biological repeats, and a total of six libraries were prepared (Nor 1, Nor 2, Nor 3, Ver 1, Ver 2, and Ver 3). After the libraries were qualified, an Illumina Novaseq 6000 (LC-BIO, China) was used for sequencing. The double-ended sequencing read length was 2 × 150 bp (PE150).

For small RNA sequencing, the TruSeq Small RNA Sample Prep Kit (Illumina) was used to prepare six small RNA sequencing libraries. After library preparation, the constructed libraries were sequenced using the Illumina HiSeq 2000/2500 (LC-BIO, China), and the sequence read lengths were 1 × 50 bp.

### mRNA identification and differential expression analysis

Readings containing undetermined bases, low-quality bases, or adapter contamination were removed using Cutadapt [67]. Sequence quality was verified using FastQC (http://www.bioinformatics.babraham.ac.uk/projects/fastqc/). According to Bowtie2 [68] and Tophat2 [69], clean reads were mapped to the reference genome of *B. rapa* v3.0, and the mapped reads were assembled with StringTie [70]. After the final transcriptome was generated, StringTie [70] and Ballgown [71] estimated the expression levels of all transcripts. DEmRNAs with | log_2_ (fold change) | > 1 and *p* ≤ 0.05 were extracted. GO terms were enriched using Blast2GO by referring to the GO database [72]. KEGG pathway analysis was carried out with reference to the KEGG pathway database (www.kegg.jp/kegg/kegg1.html) [73].

### lncRNA identification and analysis

Transcripts < 200 bp that overlapped with known mRNAs were discarded. CNCI, CPC, and Pfam were used to filter the transcripts with coding potential [74–76]. Transcripts with a CPC score < − 1 and CNCI score < 0 were deleted. The remaining class codes (i, j, o, u, and x) were considered lncRNAs, which may play a cis-acting role in adjacent target genes. The expression levels of the lncRNAs were evaluated using the fragments per kilobase of exon per million mapped reads method. Differential expression of lncRNAs was screened by | log_2_ (fold change) | > 1 and *p* ≤ 0.05. To study the function of the lncRNAs, we predicted their cis-target genes. To achieve this, Perl scripts were used to screen 100 kb upstream and downstream for coding genes. We then used internal scripts to analyze the functions of the lncRNA target genes.

### circRNA identification and analysis

Circexplorer assembled the mapping reads into circRNA [71, 75]. TopHat Fusion and Circexplorer then identified the reverse-splicing reads in the unmapped reads. Based on the structural and splicing sequence characteristics of the circRNAs, the following criteria were used for identification: (1) back-spliced junction reads ≥ 1; (2) mismatch ≤ 2; and (3) distance between the two splicing sites < 100 kb. The circRNA expression levels were calculated using an internal script. The R package edgeR was used to identify the DEcircRNAs between the Nor and Ver libraries, with a | log_2_ (fold change) | > 1 and *p* ≤ 0.05 [76].

### Identification of miRNAs and the prediction of their target genes

After filtering out low complexity, adapter dimers, common RNA families (tRNA, snRNA, sonRNA, and rRNA), and duplications in the original reads, the unique 18‒25 nucleotide sequences were mapped to specific species precursors in miRBase 21.0 (http://www.mirbase.org/) by BLAST search to identify known and novel miRNAs. We analyzed and compared the expression levels of the miRNAs between the Nor and Ver libraries. miRNAs were considered differentially expressed when the | log_2_ (fold change) | > 1 and *p* ≤ 0.05. We used the computational target finder algorithm (TargetFinder) to identify the miRNA binding sites and predict the most abundant miRNA-targeted genes. The GO terms and KEGG pathways for most miRNA targets were also annotated.

### ceRNA network construction and analysis

To reveal the interactions among the mRNAs, miRNAs, lncRNAs, and circRNAs, a ceRNA regulatory network was constructed using circRNA/lncRNA‒miRNA‒mRNA based on the ceRNA hypothesis. The miRNA‒mRNA, miRNA‒lncRNA, and miRNA‒circRNA pairs were predicted using psRobot [77]. Pairwise correlations of the miRNA‒mRNA, miRNA‒lncRNA, and miRNA‒circRNA interactions were evaluated using the Spearman correlation coefficient (SCC) and paired expression profile data [78]. The interaction network was constructed with the RNA pairs with SCC < − 0.5 and visualized using Cytoscape software (https://cytoscape.org) [79].

### Quantitative reverse-transcriptase-polymerase chain reaction (qRT-PCR) validation

To verify the reliability of the whole-transcriptome RNA sequencing results, we randomly selected four DEmRNAs, four DEmiRNAs, four DEcircRNAs, and four DElncRNAs for qRT-PCR validation. Total RNA from the Nor and Ver seed samples was extracted using a TRIzol kit (Invitrogen, USA). RNA was reverse-transcribed into cDNA using an Evo M-MLV RT Kit II according to the manufacturer’s instructions (Accurate Biotechnology, AG11711, China). The mRNA and lncRNA were reverse-transcribed using oligo dT primers and random 6-mer primers. The miRNAs were reverse-transcribed using the specific stem-loop primers and downstream primers for the U6 endogenous reference gene. For reverse transcription of the circRNA, we used the qRT-PCR primers that we designed and the random 6-mer primers provided by the kit. The reverse-transcribed cDNA was diluted 10 times as the template for qRT‒PCR, which was performed with the SYBR Green Premix Pro Taq HS qPCR Kit according to the manufacturer’s guidelines (Accurate Biotechnology, AG11701, China) on an ABI 7300 (Thermo Fisher Scientific, Waltham, MA, USA). All qRT‒PCR reactions were performed using three technical replicates and three biological replicates. Primer sequences are listed in Table S8. RT is the specific reverse transcription primer that was used to amplify circRNAs and miRNAs. The qRT‒PCR conditions were as follows: 50 °C for 2 min, 95 °C for 30 s, followed by 40 cycles at 95 °C for 30 s, 60 °C for 30 s, and finally at 95 °C for 15 s, 60 °C for 1 min, and 95 °C for 15 s. U6 (AACGCTTCACGAATTTGCGT) was used as the endogenous control of the miRNAs. Actin (F: CGAAACAACTTACAACTCCA; R: CTCTTTGCTCATACGGTCA) was used as the endogenous control for mRNAs, lncRNAs, and circRNAs. SAS9.3 software was used to analyze the significant differences in expression levels among different samples. In addition, we detected and analyzed the expression patterns of the above-mentioned 16 RNAs at different vernalization times (0, 5, 10, 15, 20, 25, and 30 d). The qRT‒PCR method for these analyses was the same as described above.

## Supplementary information

Additional file 1 **Table S1.** Summary of the RNA-seq data.

Additional file 2 **Table S2.** Summary of all mRNAs, lncRNAs, circRNAs, and miRNAs identified in this study.

Additional file 3 **Table S3.** Summary of differentially expressed (DE) mRNAs, lncRNAs, circRNAs, and miRNAs identified in this study.

Additional file 4 **Table S4.** List of GO terms for differentially expressed (DE) mRNAs, lncRNAs, circRNAs, and miRNAs.

Additional file 5 **Table S5.** Kyoto Encyclopedia of Genes and Genome (KEGG) pathway assignments for differentially expressed (DE) mRNAs, lncRNAs, circRNAs, and miRNAs.

Additional file 6 **Table S6.** Summary of valid data from the RNA‒Seq data sRNA libraries.

Additional file 7 **Table S7.** Summary of ceRNA relationships.

Additional file 8 **Table S8.** Primers used for qRT-PCR.

## Data Availability

The datasets supporting the conclusions of this article are included within the article and its additional files. The transcriptome sequencing data were deposited in the National Center for Biotechnology Information (NCBI) Gene Expression Omnibus (GEO) Database under accession number GSE171707 and GSE171708. Genomic sequences and gene annotation information of *B. rapa* were downloaded from http://brassicadb.cn/#/.

## Abbreviations

miRNA: microRNA
lncRNA: long non-coding RNA
ceRNA: competitive endogenous RNA
FLC: flower locus C
*SOC1*: suppressor of CONSTANS overexpression 1
FT: flowering locus T
FRI: frigida
VIN3: vernalization insensitive 3
VRN1: vernalization 1
VRN2: vernalization 2
ncRNAs: non-coding RNAs
circRNAs: circular RNAs
GO: Gene Ontology
KEGG: Kyoto Encyclopedia of Genes and Genome
SCC: Spearman correlation coefficient
qRT‒PCR: quantitative real-time PCR
DEmRNAs: differentially expressed mRNAs
bHLH: basic helix-loop-helix
BP: biological process
CC: cytoplasm under cellular component
MF: molecular function
DElncRNAs: differentially expressed lncRNAs
DEcircRNAs: differentially expressed circRNAs
DEmiRNAs: differentially expressed miRNAs
ERF: ethylene-responsive transcription factor
