# Supplementary material for: Whole-transcriptome sequencing reveals a vernalization-related ceRNA regulatory network in chinese cabbage (Brassica campestris L. ssp. pekinensis)
Source: BMC Genomics. 2021 Nov 13;22:819. doi: 10.1186/s12864-021-08110-2 (PMC8590779; doi:10.1186/s12864-021-08110-2)
Supplement: Supplementary file 1 — Table S1. Summary of the RNA-seq data. [file 12864_2021_8110_MOESM1_ESM.docx]

Table S1 Summary of the RNA-seq data

| Sample | Nor1 | Nor2 | Nor3 | Ver1 | Ver2 | Ver3 |
| --- | --- | --- | --- | --- | --- | --- |
| Raw data Read | 109656584 | 101960274 | 106450504 | 100021522 | 102908868 | 105475762 |
| Raw data Base | 16.45G | 15.29G | 15.97G | 15.00G | 15.44G | 15.82G |
| Valid data Read | 91577148 | 76477304 | 92183402 | 84629688 | 85688698 | 89520912 |
| Valid data Base | 13.74G | 11.47G | 13.83G | 12.69G | 12.85G | 13.43G |
| Valid ratio | 83.51% | 75.01% | 86.60% | 84.61% | 83.27% | 84.87% |
| Q20 | 99.99% | 99.99% | 99.99% | 99.98% | 99.98% | 99.98% |
| Q30 | 97.95% | 97.92% | 97.81% | 97.87% | 97.88% | 97.84% |
| GCcontent | 45% | 45% | 45% | 46% | 46% | 46% |
| Mapped reads | 80662031(88.08%) | 67421958(88.16%) | 80988659(87.86%) | 74834584(88.43%) | 75947808(88.63%) | 79255843(88.53%) |
| Unique Mapped reads | 65119872(71.11%) | 54633581(71.44%) | 65222665(70.75%) | 60642550(71.66%) | 61400002(71.65%) | 63984481(71.47%) |
| Multi Mapped reads | 15542159(16.97%) | 12788377(16.72%) | 15765994(17.10%) | 14192034(16.77%) | 14547806(16.98%) | 15271362(17.06%) |
| Reads map to sense strand | 38451732(41.99%) | 32094129(41.97%) | 38533252(41.80%) | 35672715(42.15%) | 36238295(42.29%) | 37732605(42.15%) |
| Reads map to antisense strand | 38662060(42.22%) | 32240225(42.16%) | 38753162(42.04%) | 35766519(42.26%) | 36451020(42.54%) | 37916054(42.35%) |
| Non-splice | 59684838(65.17%) | 49836592(65.17%) | 59519443(64.57%) | 55696940(65.81%) | 56775388(66.26%) | 58972384(65.88%) |
| splice | 17428954(19.03%) | 14497762(18.96%) | 17766971(19.27%) | 15742294(18.60%) | 15913927(18.57%) | 16676275(18.63%) |
