# Supplementary material for: Whole-transcriptome sequencing reveals a vernalization-related ceRNA regulatory network in chinese cabbage (Brassica campestris L. ssp. pekinensis)
Source: BMC Genomics. 2021 Nov 13;22:819. doi: 10.1186/s12864-021-08110-2 (PMC8590779; doi:10.1186/s12864-021-08110-2)
Supplement: Supplementary file 6 — Table S6. Summary of valid data from the RNA‒Seq data sRNA libraries. [file 12864_2021_8110_MOESM6_ESM.docx]

**Table S6** Summary of valid data from the summary of the RNA‒Seq data sRNA libraries.

| Library | | Raw reads | 3ADT&length filter | Junk reads | Rfam | mRNA | Repeats | Valid reads |
| --- | --- | --- | --- | --- | --- | --- | --- | --- |
| ‘Nor1’ | Total | 18561688 | 6479154 | 77532 | 1294088 | 406260 | 76372 | 10285352 |
|  | %of Total | 100 | 34.91 | 0.42 | 6.97 | 2.19 | 0.41 | 55.41 |
|  | Unique | 3409924 | 906166 | 45034 | 19733 | 9016 | 664 | 2430404 |
|  | %of Unique | 100 | 26.57 | 1.32 | 0.58 | 0.26 | 0.02 | 71.27 |
| ‘Nor2’ | Total | 17421878 | 8030770 | 58057 | 1020033 | 314940 | 76773 | 7975575 |
|  | %of Total | 100 | 46.1 | 0.33 | 5.85 | 1.81 | 0.44 | 45.78 |
|  | Unique | 3101411 | 1045371 | 35933 | 17364 | 6792 | 588 | 1996355 |
|  | %of Unique | 100 | 33.71 | 1.16 | 0.56 | 0.22 | 0.02 | 64.37 |
| ‘Nor3’ | Total | 16806048 | 7076057 | 64813 | 1028573 | 313511 | 63628 | 8305774 |
|  | %of Total | 100 | 42.1 | 0.39 | 6.12 | 1.87 | 0.38 | 49.42 |
|  | Unique | 3147298 | 984216 | 39110 | 17394 | 7002 | 586 | 2099889 |
|  | %of Unique | 100 | 31.27 | 1.24 | 0.55 | 0.22 | 0.02 | 66.72 |
| ‘Ver1’ | Total | 18084473 | 8996195 | 45595 | 1174069 | 430239 | 26052 | 7448976 |
|  | %of Total | 100 | 49.75 | 0.25 | 6.49 | 2.38 | 0.14 | 41.19 |
|  | Unique | 2277523 | 888914 | 27631 | 16250 | 7526 | 455 | 1337723 |
|  | %of Unique | 100 | 39.03 | 1.21 | 0.71 | 0.33 | 0.02 | 58.74 |
| ‘Ver2’ | Total | 18189123 | 10149537 | 46691 | 1006249 | 308961 | 17961 | 6685516 |
|  | %of Total | 100 | 55.8 | 0.26 | 5.53 | 1.7 | 0.1 | 36.76 |
|  | Unique | 2267043 | 938522 | 28020 | 14866 | 6318 | 392 | 1279714 |
|  | %of Unique | 100 | 41.4 | 1.24 | 0.66 | 0.28 | 0.02 | 56.45 |
| ‘Ver3’ | Total | 16014864 | 8521263 | 33250 | 936210 | 314363 | 18217 | 6217776 |
|  | %of Total | 100 | 53.21 | 0.21 | 5.85 | 1.96 | 0.11 | 38.83 |
|  | Unique | 2214851 | 915203 | 21911 | 16068 | 5842 | 394 | 1256294 |
|  | %of Unique | 100 | 41.32 | 0.99 | 0.73 | 0.26 | 0.02 | 56.72 |
